# All-Printed Microfluidic–Electrochemical Devices for Glucose Detection

**DOI:** 10.3390/bios14120569

**Published:** 2024-11-24

**Authors:** Zexi Wang, Zhiyi Zhang, Changqing Xu

**Affiliations:** 1 School of Biomedical Engineering, McMaster University, Hamilton, ON L8S 4L8, Canada; 2 Quantum and Nanotechnologies Research Center, National Research Council Canada, Ottawa, ON K1A 0R6, Canada; 3 Engineering Physics, McMaster University, Hamilton, ON L8S 4L8, Canada

**Keywords:** printing, porous materials, capillary microfluidic, electrochemical, glucose sensing

## Abstract

Free-standing capillary microfluidic channels were directly printed over printed electrodes using a particle/polymer mixture to fabricate microfluidic–electrochemical devices on polyethylene terephthalate (PET) films. Printed devices with no electrode modification were demonstrated to have the lowest limit of detection (LOD) of 7 μM for sensing glucose. The study shows that both a low polymer concentration in the mixture for printing the microfluidic channels and surface modification of the printed microfluidic channels using 3-aminopropyltrimethoxysilane can substantially boost the device’s performance. It also shows that both device structure and enzyme doping level of the devices play an important role in ensuring the best performance of the devices under various testing conditions.

## 1. Introduction

Microfluidic–electrochemical devices have received growing research interest, with advanced technologies developed for the conversion and detection of chemicals. The devices allow good control of mass transport and reaction through electrochemistry or the detection of small analyte volumes with high sensitivity and selectivity [1]. Their typical applications include microfluidic fuel cells [2,3], microfluidic sensing [4,5,6], and others. Miniaturization and portability are the common features of the devices.

A microfluidic-electrochemical device is fabricated by establishing direct contact between electrodes and a microfluidic channel on a single substrate so that the fluid flowing within the channel can reach the electrodes for reaction. When fluidic distribution to several locations is required, such contact is commonly achieved by attaching microfluidic channels to the substrate with electrodes on its surface or depositing electrodes directly on the wall of a microfluidic channel. The former method is mostly used for traditional hollow microfluidic channels. In a widely used technique, a rubbery PDMS (polydimethylsiloxane) slab with microfluidic channels is chemically bonded onto a piece of glass or silicon wafer with electrodes on its surface [7,8,9]. Microfluidic slabs made from thermoplastic polymers, such as PMMA (Polymethyl methacrylate) [10] and PTFE (polytetrafluoroethylene) [11], were also mechanically attached to the substrates with electrodes on their surface. The latter is mostly used for paper-based microfluidic channels. Normally, microfluidic channels are first created using filter papers by introducing a hydrophobic barrier onto the papers; then, conductive inks are printed over the channels [4,12,13]. Conductive carbon ink [12], Ag/Ag ink [12], graphene-modified carbon ink [14], black carbon/Prussian blue nanocomposite graphite ink [15], Au nanoparticles ink [16], and others are used for depositing the electrodes using screen printing [12], stencil printing [17], and inkjet printing [18]. Other methods, such as graphite pencil drawing [19] and metal sputtering [20], are used to generate electrodes on paper-based microfluidic channels.

Each of the above methods of integrating electrodes with microfluidic channels has its strengths and drawbacks. For the first one, quality and low-cost electrodes can be used as there are many commercial processes for fabricating electrodes on smooth and dense surfaces using a minimum amount of conductive material. However, it is difficult to scale up the attachment of microfluidic slabs to a flat surface to achieve fluidic sealing and good alignment of electrodes for volume processing at a low cost. For the second method, printing electrodes directly on microfluidic channels opens the door for integration, as printing is suitable for volume production at a low cost. But, as the papers used for fabricating microfluidic devices are very porous [21], it is difficult to obtain low-resistance electrodes on paper. In principle, a large amount of conductive ink has to be put on the paper surface to achieve the targeted electric performance. Multiple printing passes [22], for example, were used for printing the electrodes on paper-based microfluidic channels, which is associated with high material and process costs. In addition, ink spread in porous papers is not in favor of controlling electrode edges and obtaining small electrode-to-electrode gaps.

Our developed technology of printed capillary microfluidic devices has introduced a new way of fabricating microfluidic–electrochemical devices, which has advantages and avoids the disadvantages of the above two methods [23]. With this technology, capillary microfluidic channels can be directly printed on a smooth and dense surface equipped with electrodes using a particle/polymer liquid mixture. The obtained free-standing or raised channels contain interconnected pores with hydrophilic surfaces and can transport aqueous solutions through capillary action in the same fashion as paper-based microfluidic channels. They are bonded to the deposition surface firmly through the polymer in the printed material and can keep the solution within the channels through their hydrophilic pores without leaking it. The technology allows the use of quality electrodes fabricated by well-established processes and the use of printing to achieve the integration of electrodes with microfluidic channels. In this paper, we report the microfluidic–electrochemical devices fabricated by printing microfluidic channels over electrodes printed on PET (polyethylene terephthalate) films. The research focus is on the fundamental aspects that can be engineered for glucose sensing with a low limit of detection (LOD).

## 2. Experimental Methods

### 2.1. Chemicals

Various commercial chemicals were purchased and used to fabricate and test the devices. Dimethyl sulfoxide (DMSO), polyvinyl alcohol (PVA), fumed silica, glucose oxidase (from Aspergillus niger, 159,579 U/g) (GOx), potassium chloride (KCl), potassium (III) ferricyanide (K_3_[Fe(CN)_6_]), dextrose, 3-aminopropyltrimethoxysilane, and phosphate-buffered saline (PBS) were purchase from Millipore Sigma (Oakville, ON, Canada). Surfactant was purchased from Dynax (Pound Ridge, NY, USA). Aluminum oxide or alumina particles were purchased from SkySpring Nanomaterials Inc. (Houston, TX, USA). Carbon ink (ECI 7001 E&C) was purchased from Loctite (Westlake, OH, USA) and silver conductor ink (5025) was purchased from Dupont (Wilmington, DE, USA). Ag/AgCl ink was purchased from Kayaku Advanced Materials Inc. (Westborough, MA, USA). Polyethylene terephthalate (PET, ST 505) films were purchased from Tekra (New Berlin, WI, USA).

### 2.2. Material Preparation and Device Fabrication

The materials for printing capillary microfluidic channels were prepared by mixing dimethyl sulfoxide (DMSO), fumed silica, PVA/DMSO solution, and alumina particles in two-step mixing described earlier [23]. A vortex mixer was used to mix the first two chemicals, and a speed mixer from FlackTek Inc. (Louisville, Co, USA) was used to homogenize the mixture with the remaining components. Two types of mixtures, containing 8% PVA and 3% PVA solid mass separately, were formulated and are referred to as the material containing 8% PVA and the material containing 3% PVA.

**Figure 1:**
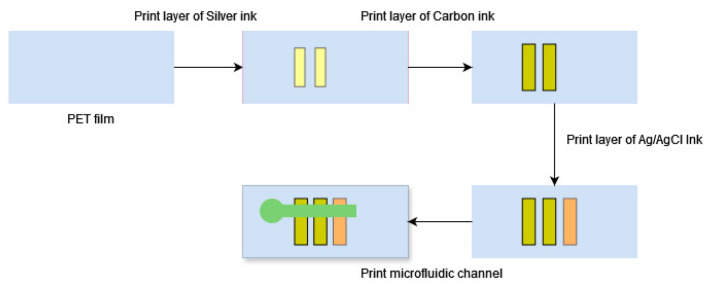
Process flow for printing capillary microfluidics devices for electrochemical sensing.

Figure 1 summarizes the process flowchart for device fabrication. Firstly, a layer of silver conductive ink and then a layer of carbon conductive inks were printed one by one to form the working and counter electrodes. Then, Ag/AgCl was printed as the reference electrode in addition to the working electrodes. Lastly, two layers of the prepared materials for microfluidic channels were printed over the electrodes. Proper alignment was adjusted for the printing of each layer, and each freshly printed layer was dried at 120 °C for 10 min.

An ASYS EKRA X1-SL semi-automatic screen printer (ASYS group, Dornstradt, Germany) was used for printing the whole device on PET films. The distance between the screen and the substrate was set to approximately 2.4 mm. The devices were printed using a 75 Shore A polyurethane squeegee with a 65° attack angle. A pressure of 1.5 bar was applied to the squeegee, and the print speed was 30 mm/s. The separation speed was set to 0.5 mm/s. The screens used include a 200-mesh steel screen for depositing the silver layer, a 200-mesh polyester screen for forming reference electrodes, and a 200-mesh steel screen for forming the working and counter electrodes.

### 2.3. Device Characterization

The all-printed devices were analyzed using a Princeton Applied Research PARSTAT 2263 potentiostat (AMETEK Scientific Instrument, Oakridge, TN, USA). In the cyclic voltammetry experiments, 6 µL of 1 mM potassium ferricyanide dissolved in 0.5 M KCl and 0.1 M PBS buffer (pH 7.4) was dropped onto the inlet of each channel and allowed to spread through the channel. The experiments were performed within the potential of 0–1 V at various scan rates ranging from 25 to 400 mV/s. The obtained anodic peak current was plotted versus the square root of the scan rate to analyze their relationship.

**Figure 2:**
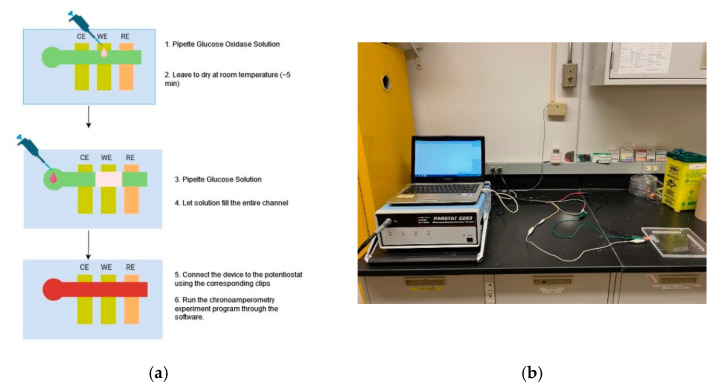
Graphic overview of the experimental procedures and setup for chronoamperometry experiments. (**a**) Schematic flowchart of the chronoamperometry experiment; (**b**) image of the experimental setup.

In the chronoamperometry experiments, electrochemical detection of glucose was conducted. The redox reaction of interest features glucose oxidase, which oxidizes glucose to gluconic acid. This oxidation was coupled with the reduction of potassium ferricyanide to potassium ferrocyanide. The ferrocyanide can then be converted back to ferricyanide at a set potential on the surface of the electrode. This conversion generates the current measured in chronoamperometry. Figure 2 shows the experimental procedures and setup for chronoamperometry analysis. Glucose samples ranging from 0 to 25 mM were prepared in 0.1 M PBS buffer at pH 7.4 and allowed to undergo mutarotation overnight. Chronoamperometry was performed using a step potential at 400 mV of the potentiostat that was connected to the devices with clips and cables. In the analysis, 1 µL of enzyme (glucose oxidase) solution (800 U/mL or 2400 U/mL) containing 100 mM potassium ferricyanide and 1 M KCl in 0.1 M PBS at pH 7.4 was first added onto each microfluidic channel over the working electrode and allowed to dry at room temperature. Then, 6 µL of the glucose sample was added to the channel inlet and allowed to travel through the whole channel (within 1 min). Measurement was performed immediately after the sample had thoroughly filled the channel, and the current measured at 30 s was used for further analysis.

In the study of improving device performance through immobilization, the pore surface of the microfluidic channels was modified before the enzyme solution was added. The modification was completed by spotting a solution with 1% 3-aminopropyltrimethoxysilane in ethanol [23] onto the detection zone of each device and heating the device at 120 °C for 10 min.

## 3. Results and Discussion

### 3.1. Device Performance

**Figure 3:**
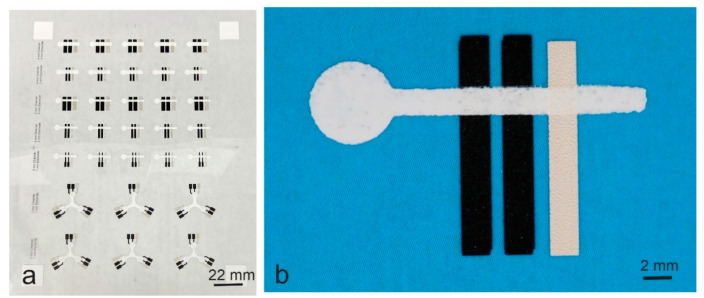
Photos of the printed microfluidic–electrochemical devices. (**a**) Multiple devices printed on a polyethylene terephthalate (PET) film using a screen printer. (**b**) A device with a 2 mm wide microfluidic channel and 2 mm wide electrodes when a blue paper was placed underneath for photo contrast. The microfluidic channel was printed with the material containing 8% PVA.

Figure 3 shows the microfluidic–electrochemical devices printed on a polyethylene terephthalate (PET) film using a screen printer as described in the Experimental Section. Working/counter electrodes (43 μm thick) and reference electrodes (45 μm thick) were first printed on the film, and microfluidic channels, 51 μm thick, were then printed over the electrodes. The design of the devices was adopted from the previously reported paper-based microfluidic electrochemical devices (called ePADs) [12,22] and was incorporated with a number of dimensions (Figure 3a). Among them, the devices with straight microfluidic channels were focused on understanding the impact of the channels on electrochemical detection. Two microfluidic channel widths, namely 2 mm and 2 mm, were included in the studies. Figure 3b shows a photo of a device in which the electrodes are 2 mm wide and the microfluidic channel is 2 mm wide.

**Figure 4:**
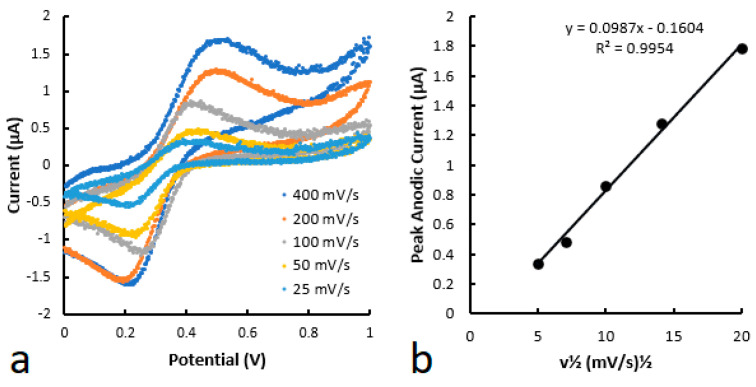
(**a**) Cyclic voltammograms of 1 mM K_3_[Fe(CN)_6_] at various voltages. (**b**) Anodic peak current plotted as a function of the square root of the scan rate.

The all-printed microfluidic-electrochemical devices, as seen in Figure 3b, were tested using cyclic voltammetry for 1 mM potassium ferricyanide dissolved in 0.5 M KCl and 0.1 M PBS buffer (pH 7.4) as shown in Figure 4. Scans at 25, 50, 100, 200, and 400 mV/s (Figure 4a) were analyzed and the obtained anodic peak current was plotted against the square root of the scan rate (Figure 4b). As demonstrated by the shape of the cyclic voltammograms (Figure 4a), the redox reaction of potassium ferricyanide is a reversible process, making it a suitable mediator for the detection of glucose. Furthermore, the linearity of Figure 4b indicates that the analytes follow the Randles-Sevcik equation, and the potassium ferricyanide can freely diffuse within the printed microfluidic channel [24]. These results are in agreement with the previous study, which used the same method with ePADs [22]. As explained previously, glucose oxidase catalyzes the oxidation of glucose into gluconic acid with the reduction of potassium ferricyanide to potassium ferrocyanide as a co-product [25]. The electrochemical oxidation back to ferricyanide can then be measured as a current using chronoamperometry [22].

**Figure 5:**
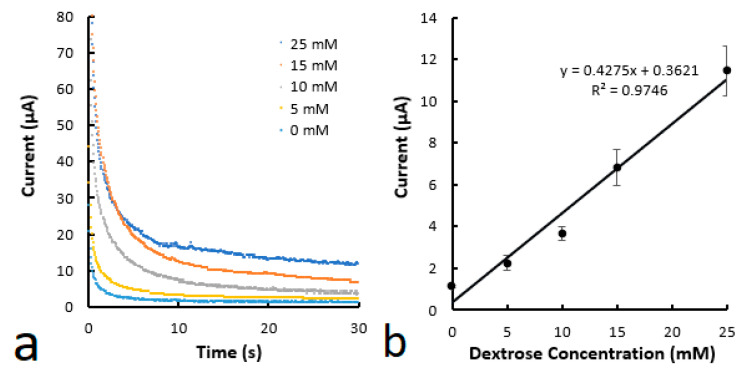
(**a**) Chronoamperometric curves for various glucose concentrations. (**b**) Calibration curve of glucose concentration as a function of current at 30 s. The microfluidic channels were printed using the material containing 8% PVA.

Figure 5 shows the chronoamperometric measurement of the devices when solutions with various glucose concentrations are applied. The current over time was recorded for each concentration (Figure 5a), and the observed current at 30 s under each concentration was used to create a calibration curve (Figure 5b). The calibration curve shows that the current is linearly proportional to the glucose concentration in the tested range. A limit of detection (LOD) of 0.24 mM was determined following the method previously reported [26]. This LOD value was comparable to the value of 0.21-0.35 mM of the various ePADs reported [12,22]. These reported ePADs were fabricated by creating microfluidic channels on paper through photolithography and printing conductive carbon and Ag/AgCl electrodes over the papers through multiple passes.

The all-printed microfluidic–electrochemical devices are fabricated by printing to establish the contact of electrodes with microfluidic channels. A strong bonding is naturally formed through the polymer in the printed materials after the involved solvent is evaporated. This integration method is much simpler than that of attaching a microfluidic slab to an electrode-ready surface and is suitable for volume processing at a low cost. It is also different from that of printing electrodes onto paper, as described in the Introduction Section, resulting in a structure different from paper-based devices (i.e., ePADs). In the all-printed ones, the electrodes lay on PET films and the free-standing microfluidic channels sit over the electrodes and the PET films, which serve as protective substrates [23]. In the latter ones, microfluidic channels are embedded in the paper and electrodes sit on the paper. In comparison with the fabrication of ePADs, printing electrodes on smooth PET films and integrating them with the printed microfluidic channels is more suitable for volume production. Firstly, low-resistance electrodes can be easily printed in a single pass using commercial conductive carbon inks and Ag/AgCl inks. Secondly, well-defined electrodes with sharp edges and small electrode-to-electrode gaps at 150-200 μm can be repeatedly printed, in high volume using screen printing, on polymer films [27]. Thirdly, fine alignment can be easily achieved to ensure precise integration of each involved component because fine alignment marks can be easily printed on PET films, which are rigid enough for fast handling in both sheet-to-sheet and roll-to-roll printing. For ePADs, as the typical filter papers used for microfluidic devices are very porous and the pores are hydrophilic, in principle, it is difficult to print low-resistance electrodes on the papers using minimum amounts of conductive inks. It is also difficult to control electrode edges and obtain small electrode-to-electrode gaps due to ink spreading on the papers, and it is difficult to create high-resolution alignment marks on the papers for the accurate alignment required in volume production. Meanwhile, the all-printed devices can handle much smaller sample volumes than the ePADs. A 6 μL volume of the testing sample was used for the device shown in Figure 3b, while 100 μL was required by ePADs with a similar device structure [22].

### 3.2. Impact of Microfluidic Materials

The above LOD at 0.24 mM can be substantially reduced by engineering the materials that form the capillary microfluidic channels. As described in the Experimental Section, the materials used for printing the capillary microfluidic channels contain particles, polymer binder PVA, and solvent. It is believed that PVA may have a negative impact on the device’s performance. Firstly, the PVA dissolved in the materials may accumulate on the electrode surface after the materials are printed over the electrodes. The reason is that the PVA solvent solution flows and can easily sink from its mixture with particles. As all conventional polymers, including PVA, are electrical insulators [28], the PVA accumulated on the electrode surface naturally serves as an insulation layer that reduces electronic conduction. Secondly, the pores in the microfluidic channels are formed by the packing voids in the particles, and the PVA solution may flow into the voids, with PVA being trapped there after the solvent is evaporated. This part of PVA naturally reduces the open space in the packing voids, which reduces the fluidic flow and fluidic volume within the channels.

With this understanding, the PVA concentration in the materials used for printing the devices was minimized. After testing the materials with various PVA concentrations, it was concluded that 3% PVA was the lowest level required for acceptable printing performance and integrity of the printed structure. The devices printed with microfluidic materials containing 3% PVA were determined to have an LOD of 0.077 mM for glucose detection. This is a substantial decrease from the 0.25 mM for devices printed with the material containing 8% PVA. It supports the initial hypothesis that PVA may act as an electrical insulator on electrode surfaces and may reduce the porosity of the microfluidic channels. By lowering the PVA concentration in the material, the negative effect of the polymer is reduced, leading to a significant improvement in device performance.

**Figure 6:**
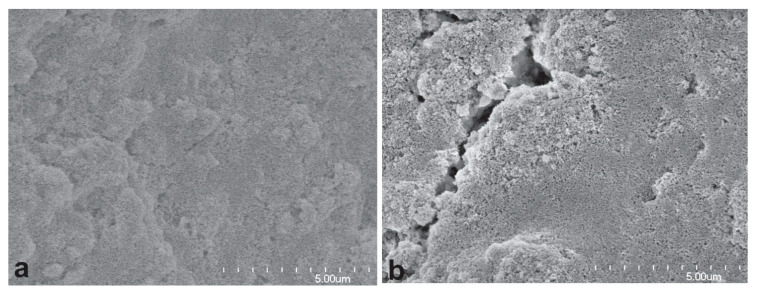
SEM images of the microfluidic channels. (**a**) Printed with the material containing 8% PVA; (**b**) printed with the material containing 3% PVA.

The impact of PVA on the porous structure of the printed microfluidic channels was confirmed by SEM analysis; however, the impact of PVA on electrical conduction is difficult to isolate and test. It was observed, under SEM, that the microfluidic channels printed using the material containing 8% PVA (Figure 6a) are less porous than those printed with the ones with 3% PVA (Figure 6b). The pore sizes of the former are also smaller than those of the latter.

**Figure 7:**
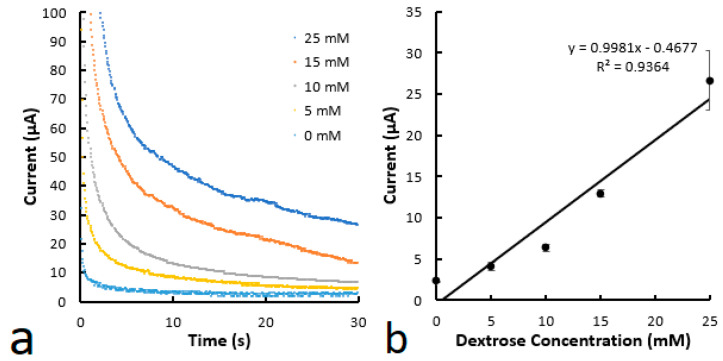
(**a**) Chronoamperometric curves for various glucose concentrations. (**b**) Calibration curve of glucose concentration as a function of current at 30 s. The microfluidic channels were printed using the material with 3% PVA for the first layer and the material with 8% PVA for the second layer. The device structure is the same as that in Figure 3b.

Unfortunately, the microfluidic channels printed using the material containing 3% PVA are less uniform than those printed with the ones containing 8% PVA and there are small cracks in the channels printed using the lower PVA-containing material. This poorer film uniformity and cracks are associated with higher noise in the chronoamperometric curves of the devices in comparison with the devices containing 8% PVA. A possible reason is that the defects cause a less uniform distribution of the doped enzyme in the channels. Also, the scratch resistance of the printed channels is decreased when the PVA content is reduced from 8% to 3% because there is less PVA to hold the particles. To overcome the above shortcomings, a new microfluidic channel with the 3% PVA material as the first layer and the 8% PVA material as the second layer was proposed. The goal of the hybrid model was to use the material with a higher PVA to cover the one with a lower PVA to improve its film uniformity and scratch resistance. The printed microfluidic channels with the hybrid structure were shown to have similar film uniformity and scratch resistance as the devices printed with the 8% PVA material only. The reason is that the 8% PVA material can reflow on the films printed with 3% PVA material to fill the defective areas and cracks to form a protective shell. Chronoamperometry was conducted with the printed microfluidic devices (Figure 7), and a similar LOD of 0.081 mM was obtained. The noise level in these hybrid devices is improved to the level observed for the devices fabricated using the 8% PVA material. This shows that the hybrid approach is effective in overcoming the shortcomings of the devices printed with less PVA material while retaining their sensitivity.

### 3.3. Immobilization Effect

**Figure 8:**
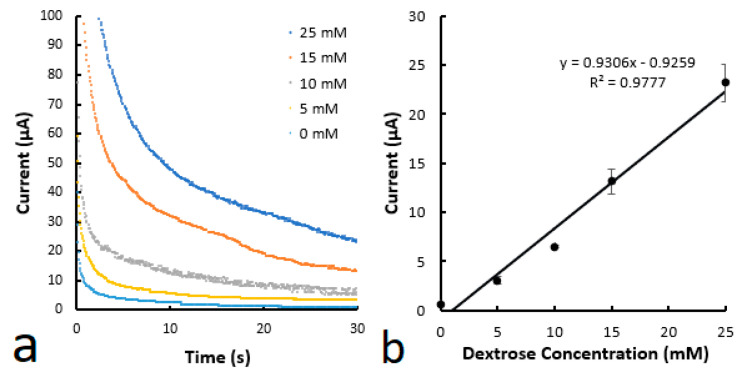
(**a**) Chronoamperometric curves for various glucose concentrations. (**b**) Calibration curve of glucose concentration as a function of current at 30 s. The microfluidic channels were printed using the mixture with 3% PVA for the first layer and the material with 8% PVA for the second layer. The device structure is the same as that in Figure 3b. An ethanol solution of 1% 3-aminopropyltrimethoxysilane was applied to the detection zone and dried at 120 °C.

In the enzyme-based electrochemical detection of glucose, the enzyme needs to be applied to the detection zone of the microfluidic channel, as illustrated in Figure 2. The applied enzyme, in this case, is exposed to a risk of being washed away by the glucose solution that flows through the microfluidic channel from the inlet zone. A similar enzyme-washing effect, which is associated with uneven color change, was observed in colorimetric glucose detection in paper-based microfluidic devices [28] and in printed capillary microfluidic devices [23]. In electrochemical detection, this washing effect could cause reproducibility issues between measurements as the enzyme concentration is not static. In our previous research on colorimetric glucose detection, 3-aminopropyltrimethoxysilane was applied to modify the pore surface of the printed microfluidic devices [23]. It was found that surface modification was effective in immobilizing the enzyme and improving color uniformity. This method was thus applied to the printed microfluidic–electrochemical devices in this work. In the application, a 1% 3-aminopropyltrimethoxysilane solution in ethanol was first dropped into the detection zone and dried before the enzyme was doped. Figure 8 shows the results of the hybrid devices treated with the process. The LOD from the test was calculated to be 0.023 mM, which is a substantial improvement from the above 0.081 mM.

3-aminopropyltrimethoxysilane is widely used in surface modification for biosensing as it contains three hydrolysable substituents that react with up to three OH groups on the alumina and silica particles within the microfluidic channels and an amine group that can interact with an enzyme molecule. By modifying the pore surface of the microfluidic channels with the chemical, the applied enzyme is apparently immobilized on the pore surface to a certain degree to withstand the flow-caused washing effect of the glucose solution, allowing more enzyme to be used for electrochemical detection. This shows that the method developed for colorimetric glucose detection [23] is effective for electrochemical detection. This immobilization step can be added to the process of device fabrication without changing the nature of the devices such that the whole microfluidic–electrochemical devices are printed with a single screen printer in a process that is suitable for low-cost and mass production.

### 3.4. Testing Conditions and Device Structure

While the above immobilization process proved effective in decreasing the washing effect caused by the sample flow, it does not completely eliminate it, and the reduced washing effect is still visible in some special experiments. In one case, when the test sample volume was decreased from 6 μL to 4 μL for the above immobilized hybrid devices, the obtained device-to-device testing variation that is associated with the standard deviation for LOD calculation [26] was lowered, resulting in a reduction in the corresponding LOD from 0.023 to 0.012 mM. This indicates that there is still a washing effect in the above tests when using 6 μL of the sample, and the effect is reduced by the decreased sample flow when the sample volume is 4 μL. In another case, when the microfluidic channel width of the above device was increased from 2 mm to 4 mm, an LOD of 0.012 was still obtained for the 6 μL sample volume. This increase in channel width directly expands the cross-section of the microfluidic channel, resulting in decreased sample flow through each pore and, thus, the washing effect.

**Figure 9:**
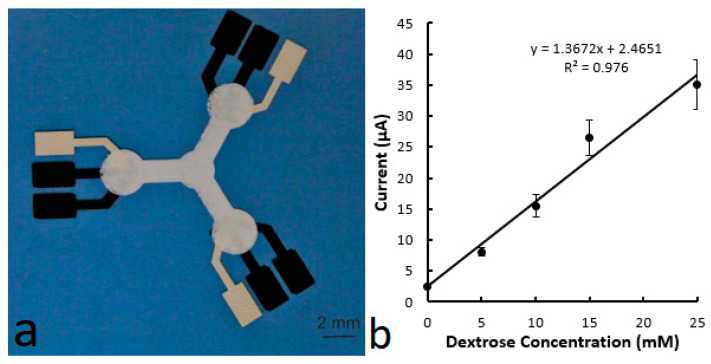
(**a**) Photo of a printed microfluidic–electrochemical device. (**b**) Calibration curve of glucose concentration as a function of current at 30 s. The microfluidic channels were printed using the material with 3% PVA for the first layer and the mixture with 8% PVA for the top layer. An ethanol solution of 1% 3-aminopropyltrimethoxysilane was applied to the detection zone and dried at 120 °C.

The washing effect can be further reduced by designing a different device structure and optimizing the immobilization process. In the studied devices (as seen in Figure 3b), the washing effect is maximized as the test sample flows through the detection zone to the channel end. This situation is improved when the detection zone is located at the end of the microfluidic channel. Figure 9 shows a device printed simultaneously with the above hybrid devices on the same PET film (as seen in Figure 3a). The device structure is adopted from reported ePADs for testing three analytes [12]. To test the washing effect, the three microfluidic channels were cut off at the inlet (central cycle) edge and then treated with the same 3-aminopropyltrimethoxysilane solution. When 2 or 4 μL of the testing sample was dropped from the cutting end, an LOD of 0.012 mM was obtained. In this case, there is no constant sample flow to move the doped enzyme away from the detection zone.

Further research is required to improve the immobilization process to completely eliminate the washing effect. The reaction of 3-aminopropyltrimethoxysilane on the alumina surface could be enhanced by controlling the hydrolysis of the chemical and its reaction condition on the alumina surface. A proper catalyst system should be focused on in future research.

Under the condition that the enzyme washing effect is not completely eliminated and the device structure shown in Figure 3b is maintained, it was found that applying more enzyme to the detection zone is a simple way of dealing with the remaining washing effect. Table 1 shows the effects of enzyme concentration in the solution that is loaded into the detection zone. When the enzyme concentration is increased from 800 U/mL to 2400 U/mL and the testing sample volume is 6 µL, the obtained LOD is decreased from 0.012 mM to 0.007 mM. The LOD only changes to 0.008 mM if the sample volume is increased to 8 µL. When the enzyme concentration is increased to 4000 U/mL, an LOD of 0.007 mM is maintained even when the sample volume is increased to 8 µL or 10 µL.

More enzyme is applied to ensure that there is sufficient enzyme remaining in the detection zone for reaction with glucose even when washing happens. With this approach, the same LOD of 0.007 mM can be obtained even if the sample volume is more than doubled. This LOD value is the best performance obtained from testing the all-printed microfluidic-electrochemical devices. It comfortably covers all ranges of glucose concentrations found in physiological fluids such as urine, blood, sweat, and saliva [29]. By combining this approach with the optimal device structure discussed earlier, it is feasible to fabricate devices that can maintain their low LODs for a wide range of sample volumes and are therefore suitable for point-of-care applications.

The printed microfluidic-electrochemical devices are equipped with both a fluidic handling function and an electrochemical sensing function. While a single microfluidic channel can be used for receiving the test sample and delivering it to a single detection zone (as seen in Figure 3b) by capillary action, a network of microfluidic channels can be used to receive the test sample and distributing it to multiple detection zones (as seen in Figure 9) by capillary action. All the materials used for fabricating the devices are commercial, off-the-shelf, and low-cost, and the involved semi-automatic printing processes can be easily scaled up for volume production. This study has demonstrated that printing capillary microfluidic channels on electrodes deposited on a smooth and dense surface is a simple, low-cost, and scalable way of integrating electrodes with microfluidic channels. This advantage is further supported by the testing results that an LOD of 0.007 mM or 7 μM for glucose detection can be obtained for the devices using bare carbon and Ag/AgCl electrodes without any chemical modification.

The method for fabricating all-printed microfluidic-electrochemical devices is different from that used for fabricating ePADs, as described in the Introduction Section, although both types of devices use porous material to transport fluid to electrode surfaces through capillary action. In addition, the porous material and pore structure of the microfluidic channels totally differ between the two. In the channels of the former, the pores are formed by the packing voids of microparticles of alumina with alumina on the pore surface, and most pores are less than 100 nm in diameter (as seen in Figure 6) [23]. In the ePAD channels, the pores are formed by the stacking voids of cellulose fibers with cellulose on the pore surface, and the typical pores are several micrometers wide [30]. The small pores with alumina as the surface and direct contact of the alumina particles with the electrodes in the printed channels are suggested to have a positive contribution to the obtained low LOD because bare and unmodified carbon electrodes are not capable of reaching μM scale LOD during glucose detection. In our comparison, the bare electrodes (in Figure 9) without the microfluidic channels only achieved a low LOD of 0.23 mM even when immobilization and high enzyme concentration were applied. The described performance improvement through lowering PVA concentration from 8% to 3% supports this hypothesis as more pores are generated, leading to more direct contact between alumina particles and electrodes. This is also in line with some reports in the literature, in which the electrodes coated with nano-porous hydrogel films [31] and metal oxide nanoparticles [32] showed low LOD for glucose detection. While it is not clear how the pores and alumina particles would affect electrochemical detection, it is important to know that the printed microfluidic channels may serve as a functional material on electrodes to enhance detection while delivering fluidic samples to electrode surfaces through capillary action. This function is essential in achieving high performance at minimum device cost, which is required for disposable applications. In comparison, no report was found to show that cellulose filter papers can substantially improve the performance of carbon electrodes for glucose detection.

## 4. Conclusions

Capillary microfluidic channels were printed using a particle/polymer mixture over printed electrodes to fabricate microfluidic-electrochemical devices for glucose detection. The all-printed devices can perform similar functions as typical paper-based devices, and their performance can be substantially improved by printing double-layered microfluidic channels using a 3% polymer mixture for the under layer and an 8% polymer mixture for the top layer. Device performance can be further substantially improved by modifying the pore surface of the microfluidic channels with 3-aminopropyltrimethoxysilane to immobilize the applied enzyme within the detection zone. The immobilization is effective in significantly reducing sample flow-caused enzyme washing and can be combined with the use of a higher dose of enzyme to achieve the best performance. Device structure can also be designed to reduce enzyme washing. By combining the above methods, a limit of detection (LOD) of 7 μM was obtained for the final devices.

## Figures and Tables

**Table 1:** Impact of enzyme concentration on the testing performance of the devices *.

Enzyme Concentration (U/mL)	Sample Volume of Glucose Solution (µL)	LOD (mM)
800	4	0.012
800	6	0.023
2400	6	0.007
2400	8	0.008
2400	10	0.011
4000	8	0.007
4000	10	0.007

* The microfluidic channels were printed using the material with 3% PVA for the first layer and the mixture with 8% PVA for the top layer. An ethanol solution of 1% 3-aminopropyltrimethoxysilane was applied to the detection zone and dried at 120 °C.

## Data Availability

Data are contained within the article.
